# Immuno-Diagnosis of Active Tuberculosis by a Combination of Cytokines/Chemokines Induced by Two Stage-Specific Mycobacterial Antigens: A Pilot Study in a Low TB Incidence Country

**DOI:** 10.3389/fimmu.2022.842604

**Published:** 2022-03-10

**Authors:** Violette Dirix, Philippe Collart, Anne Van Praet, Maya Hites, Nicolas Dauby, Sabine Allard, Judith Racapé, Mahavir Singh, Camille Locht, Françoise Mascart, Véronique Corbière

**Affiliations:** ^1^ Laboratory of Vaccinology and Mucosal Immunity, Université Libre de Bruxelles (U.L.B.), Brussels, Belgium; ^2^ Biostatistiques du Pôle Santé (BIOPS), Université Libre de Bruxelles (U.L.B.), Brussels, Belgium; ^3^ Clinique des maladies infectieuses et tropicales, Hôpital Erasme, Université Libre de Bruxelles (U.L.B.), Brussels, Belgium; ^4^ Department of Infectious Diseases, Centre Hospitalier Universitaire Saint-Pierre, Université Libre de Bruxelles (U.L.B.), Brussels, Belgium; ^5^ Institute for Medical Immunology, Université Libre de Bruxelles (U.L.B.), Brussels, Belgium; ^6^ Dienst Interne Geneeskunde - Infectiologie, Universitair Ziekenhuis Brussel (UZ Brussel), Vrije Universiteit Brussel (VUB), Brussels, Belgium; ^7^ Biomedical Research Center, Erasme Hospital, Brussels, Belgium; ^8^ Lionex Diagnostics and Therapeutics, Braunschweig, Germany; ^9^ Univ. Lille, CNRS, Inserm, CHU Lille, Institut Pasteur de Lille, U1019 – UMR9017 – CIIL – Center for Infection and Immunity of Lille, Lille, France

**Keywords:** active tuberculosis, ESAT-6, HBHA, IFN-γ, IL-2, GM-CSF, IP-10

## Abstract

Active tuberculosis (aTB) remains a major killer from infectious disease, partially due to delayed diagnosis and hence treatment. Classical microbiological methods are slow and lack sensitivity, molecular techniques are costly and often unavailable. Moreover, available immuno-diagnostic tests lack sensitivity and do not differentiate between aTB and latent TB infection (LTBI). Here, we evaluated the performance of the combined measurement of different chemokines/cytokines induced by two different stage-specific mycobacterial antigens, Early-secreted-antigenic target-6 (ESAT-6) and Heparin-binding-haemagglutinin (HBHA), after a short *in vitro* incubation of either peripheral blood mononuclear cells (PBMC) or whole blood (WB). Blood samples were collected from a training cohort comprising 22 aTB patients, 22 LTBI subjects and 17 non-infected controls. The concentrations of 13 cytokines were measured in the supernatants. Random forest analysis identified the best markers to differentiate *M. tuberculosis-*infected from non-infected subjects, and the most appropriate markers to differentiate aTB from LTBI. Logistic regression defined predictive abilities of selected combinations of cytokines, first on the training and then on a validation cohort (17 aTB, 27 LTBI, 25 controls). Combining HBHA- and ESAT-6-induced IFN-γ concentrations produced by PBMC was optimal to differentiate infected from non-infected individuals in the training cohort (100% correct classification), but 2/16 (13%) patients with aTB were misclassified in the validation cohort. ESAT-6-induced-IP-10 combined with HBHA-induced-IFN-γ concentrations was selected to differentiate aTB from LTBI, and correctly classified 82%/77% of infected subjects as aTB or LTBI in the training/validation cohorts, respectively. Results obtained on WB also selected ESAT-6- and HBHA-induced IFN-γ concentrations to provided discrimination between infected and non-infected subjects (89%/90% correct classification in the training/validation cohorts). Further identification of aTB patients among infected subjects was best achieved by combining ESAT-6-induced IP-10 with HBHA-induced IL-2 and GM-CSF. Among infected subjects, 90%/93% of the aTB patients were correctly identified in the training/validation cohorts. We therefore propose a two steps strategy performed on 1 mL WB for a rapid identification of patients with aTB. After elimination of most non-infected subjects by combining ESAT-6 and HBHA-induced IFN-γ, the combination of IP-10, IL-2 and GM-CSF released by either ESAT-6 or HBHA correctly identifies most patients with aTB.

## Introduction

Tuberculosis (TB) remains a leading cause of death in the world, responsible for high morbidity and mortality worldwide with about 10 million new cases in 2020 and 1.5 million deaths (1). Diagnosis and hence appropriate treatment are often delayed due to the wide spectrum of clinical manifestations of active TB (aTB) and to the non-availability of sensitive and specific tests providing a rapid and accurate diagnosis (2, 3). In addition to classical clinical manifestations of aTB, this disease may occur as subclinical TB without suggestive symptoms, or as extrapulmonary TB often pauci-symptomatic in immunocompromised individuals, so that diagnosis strictly based on clinical signs or symptoms is illusive (2). The classical diagnostic method remains the identification of *M. tuberculosis* by direct smear microscopy or by culture that are both low in sensitivity and/or slow. Early diagnosis of infectious cases by sputum microscopy is only possible in approximately 50% of cases. Moreover, people with subclinical TB may likely be missed if TB culture is not performed and this is often the case in asymptomatic individuals. Molecular techniques such as GeneXpert are more sensitive but they are costly and often unavailable in primary-care settings.

Immuno-diagnosis was therefore identified as a promising approach for diagnosis of aTB. However, the commercially available tests, the interferon-γ-release assays (IGRA), based on the release of IFN-γ by blood cells in response to their *in vitro* stimulation with mycobacterial peptides corresponding to antigens encoded in the genomic region of difference (RD)-1 (the early-secreted-antigenic-target-6 (ESAT-6), and the culture-filtrate-protein-10 (CFP-10)), have a relatively high false negative rate in patients with aTB (4–6). In addition, these IGRAs, initially developed to diagnose latent TB infection (LTBI), are positive both in LTBI and in patients with aTB (7). The differential diagnosis of LTBI and aTB is therefore not possible with commercial IGRAs. New generation IGRAs were developped to improve the diagnosis of aTB. They are based either on recent studies indicating that the *M. tuberculosis* specific CD8^+^ T cell responses are positively correlated with the bacterial load and recent exposure to *M. tuberculosis* for the QuantiFERON-TB Gold Plus, or by inclusion of L-alanine dehydrogenase as an additional antigen for the LIOFeron TB/LTBI (8, 9). The added value of these improvements for the diagnosis of aTB and its differential diagnosis with LTBI was however not confirmed until now (8).

Therefore, several studies aimed to identify other proteins from *M. tuberculosis* as potential candidates to distinguish aTB from LTBI and they often also extend the cytokine measurements beyond IFN-γ to improve differential diagnosis. These studies generally first identify markers of *M. tuberculosis* infection, before applying one or several biomarkers to differentiate aTB from LTBI. Among the numerous proteins evaluated, the mycobacterial heparin-binding haemagglutinin (HBHA, Rv0475) appears as one of the most promising antigens to differentiate LTBI from aTB, but only few studies assessed HBHA-induced cytokines other than IFN-γ (10–12). In contrast, the potential added value of various cytokines induced by the peptides of the commercial IGRAs has been investigated, but with conflicting outcomes, as highlighted by a recent meta-analysis (13).

We previously reported on a combined HBHA- and ESAT-6-IGRA performed on either peripheral blood mononuclear cells (PBMC) or whole blood (WB) to diagnose LTBI and to partially differentiate LTBI from aTB (14–16). Even though this combined IGRA provided a better discrimination between LTBI and aTB than any other available *in vitro* test, it remained imperfect. In this study, we therefore measured cytokines/chemokines other than IFN-γ released in response to HBHA and to ESAT-6 and evaluated the potential added value of a combined analysis of cytokine/chemokine secretion for the differential diagnosis and especially for the diagnosis of aTB.

## Material and Methods

### Ethics Statement

The study protocol P2011/113 was approved by the ethics committee of ULB-Hôpital Erasme, Brussels, Belgium, and informed written consent was obtained from all participants.

### Study Protocol

A panel of 13 different chemokines and cytokines induced by two different mycobacterial antigens after a short-term *in vitro* stimulation of blood cells was measured. Results obtained by *in vitro* stimulation of PBMC were analyzed in parallel with those obtained by stimulating diluted WB. The two antigens, HBHA and ESAT-6, were selected as they are secreted at different stages of the mycobacterial metabolism, in order to cover the whole spectrum of *M. tuberculosis* infection (17, 18). A training cohort was constituted to select the most promising cytokines or chemokines and their best combination to differentiate infected from non-infected subjects and then to identify patients with aTB among infected individuals. The accuracy of the selected combinations was further evaluated on samples from an independent validation cohort.

### The Training and the Validation Cohorts

To ensure the reproducibility of the results and to limit the influence of a possible inclusion bias, two independent cohorts were evaluated. The training or discovery cohort comprised 61 individuals prospectively enrolled as being potentially *M. tuberculosis* infected (LTBI or aTB) or not (non-infected controls). For most of them (n=51), residual supernatants from stimulated PBMC or WB used in a previous study were used (16). Ten additional individuals (six controls, three LTBI, one aTB) were newly included. Samples from this cohort allowed us to identify the most suitable markers to first differentiate infected from non-infected subjects and then to differentiate aTB from LTBI subjects. These findings were then confirmed in a validation cohort that was independent from the training cohort and comprised 69 subjects included in a previous study (16). Residual supernatants from this cohort were used to confirm the diagnostic performance of the best combinations of markers identified on the training cohort (**Table 1**).

**Table 1:** Demographic and clinical data from the training and validation cohorts.

	aTB patients		LTBI individuals		Non-infected controls	
	Training cohort	Validation cohort	Training cohort	Validation cohort	Training cohort	Validation cohort
N	22	17	22	27	17	25
Median age (range) (yrs)	33 (19-60)	40 (18-63)	31 (21-64)	49° (19-64)	35 (21-60)	43 (21-61)
Male (no. [%])	16* (73)	9** (56)	8 (36)	12 (44)	10 (59)	5°° (20)
Ethnic origin (no. [%])						
Caucasian	8 [36]	3 [18]	13 [59]	19 [59]	16 [94]	24 [96]
North African	6 [27]	7 [41]	3 [14]	6 [19]	1 [6]	1 [4]
Sub-Saharan African	6 [27]	6 [35]	4 [18]	6 [19]	0 [0]	0
Other	2 [9]	1 [6]	2 [9]	1 [3]	0 [0]	0
Clinical data						
Pulmonary TB (no. [%])	14 [64]	12 [71]	NA	NA	NA	NA
Extrapulmonary TB (no. [%])	8 [36]	5 [29]	NA	NA	NA	NA
Positive sputum smear/culture/PCR (no. [%])	19 [86]	16 [94]	NA	NA	NA	NA

N, number; LTBI, Latent Tuberculosis Infection; aTB, active Tuberculosis; NA, not applicable.

°p=0.0014 vs LTBI subjects from the training cohort; *p=0.0329 versus LTBI subjects from the same cohort; **p=0.0229 versus controls from the same cohort °°p=0.0202 vs CTRL subjects from the training cohort.

For both cohorts, the individuals were classified in three different groups, non-infected controls, LTBI subjects and patients with aTB, based on classical criteria as reported (19). Briefly, both non-infected controls and LTBI subjects were healthy with a negative or positive (induration size of 10 mm in case of risk factor and of 15 mm for the others) tuberculin skin test (TST) respectively. LTBI subjects also had a chest radiograph with no signs of aTB infection. Active TB diagnosis was based on microbiological proof for most patients, comprising both pulmonary and extrapulmonary aTB (**Table 1**). Based on these criteria, the training cohort comprised 17 non-infected controls, 22 LTBI subjects and 22 aTB patients, whereas the validation cohort comprised 25 non-infected controls, 27 LTBI subjects and 17 aTB patients. The main demographic data of the subjects included in this study for the two cohorts are reported in **Table 1**. Differences between the median ages and the sex ratios of the individuals included in the two cohorts were minor (**Table 1**). The proportion of subjects originating from endemic countries was lower for controls than for infected individuals (p ≤ 0.05) and was the most elevated among aTB patients (**Table 1**).

### Induction of Chemokine and Cytokine Secretions

PBMC and diluted WB were *in vitro* stimulated as reported elsewhere (16, 19). Briefly, 1.10^6^ PBMC, suspended in 500 μl culture medium (RPMI with 1 ng/ml IL-7), or 250 μl WB diluted 1:1 in IL-7-enriched-AIMV medium, were incubated during 24hrs at 37°C (5% CO_2_) with HBHA purified from *Mycobacterium bovis* BCG as described (20) (1 μg for PBMC and 2 μg for WB) or with recombinant ESAT-6 (2.5 μg) provided by Lionex (Diagnostics & Therapeutics GmbH, Braunschweig, Germany), before supernatant collection. Cells incubated in antigen-free medium and incubated with staphylococcal enterotoxin B (SEB, Sigma-Aldrich, Bornem, Belgium; 0.5 and 1 μg/ml for the PBMC and diluted WB stimulation, respectively) served as negative and positive controls, respectively.

### Chemokine and Cytokine Concentration Measurements

The concentrations of 13 chemokines or cytokines were measured by multiparameter-based immunoassays (Milliplex human cytokine/chemokine kits-Merck, Belgium) according to the manufacturer’s instructions: granulocyte macrophage colony-stimulation factor (GM-CSF), Growth related oncogene (GRO), IFN-γ, interleukin (IL)-1β, IL-2, IL-6, IL-8, IL-10, IL-17A, IFN-γ-induced protein 10 (IP-10), Macrophage inflammatory protein (MIP)-1α, soluble CD40 ligand (sCD40L), and Tumor necrosis factor-alpha (TNF-α). Culture supernatants were diluted using dilution factors specific to each analyte in order to obtain concentrations within an interpretable range. Results were analysed with a Bio-Plex^®^ MAGPIX™ Multiplex reader, Bio-Plex Manager™ MP Software and Bio-Plex Manager 6.1 Software (BIO-RAD laboratories, Nazareth Eke, Belgium). If detectable, the analyte concentrations obtained in the antigen-free conditions were subtracted from those obtained with antigen stimulation. To allow statistical analyses with continuous variables, the concentrations below the detection limit were allocated the arbitrary value of half of the threshold of detection, whilst results exceeding the assay’s upper limit of detection were attributed the concentration corresponding to this limit. The laboratory scientist performing the sample analysis was blinded to the clinical and other laboratory data.

### Statistical Analysis

Differences in the concentrations of chemokines/cytokines between groups of subjects were analysed using the Mann-Whitney U test. The diagnostic abilities of individual parameters were first assessed by receiver operator characteristics (ROC) curve analysis and the areas under the curve (AUC) were calculated (GraphPad Prism version 7.03, GraphPad Software, La Jolla California USA, www.graphpad.com). The host marker selection was further evaluated by random forest analysis (random Forest package version 4.6-14) and the predictive abilities of combinations of markers were investigated by logistic regression using R (R-4.0.3, R Foundation for Statistical Computing, Vienna, Austria). Results were graphically represented with the linear predictor of the logistic regression. The tests were considered statistically significant when the *p*-value was < 0.05.

## Results

### Selection of PBMC-Produced Markers to Identify *M. tuberculosis-*Infected Subjects

We first investigated the ability of an individual marker, or a combination of markers, to differentiate *M. tuberculosis*-infected from non-infected individuals in the training cohort using ESAT-6 and HBHA for *in vitro* stimulation. Cytokine/chemokine concentrations were generally significantly higher for infected than non-infected subjects (**Supplementary Table 1**). The diagnostic accuracy of each individual marker was assessed by ROC curve analyses and most AUCs were higher than 0.7. We then performed random forest analyses including the 13 analytes secreted in response to ESAT-6 and HBHA to select the optimal combination to differentiate infected from non-infected subjects. The best discrimination was obtained by HBHA-induced IFN-γ, GM-CSF and IL-2, combined with ESAT-6-induced IFN-γ and IL-8 (**Supplementary Figure 1A**).

**Figure 1:**
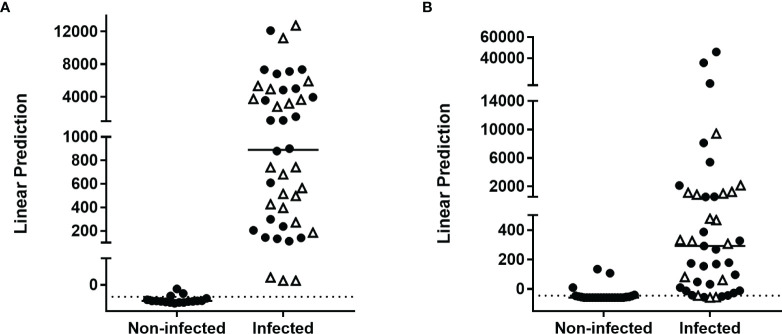
Combination of *M. tuberculosis*-specific immune markers allowing the distinction between *M. tuberculosis*-infected versus non-infected subjects in the PBMC assay. HBHA-IFN-γ and ESAT-6-IFN-γ were the markers selected by logistic regression analysis to be combined for the PBMC-based assay to discriminate *M. tuberculosis*-infected from non-infected subjects. Patients with aTB are indicated by open triangles. Results are represented with their linear predictor for the validation **(A)** and training **(B)** cohorts. The horizontal lines represent the medians and the dotted lines arbitrary cut-offs.

**Figure 2:**
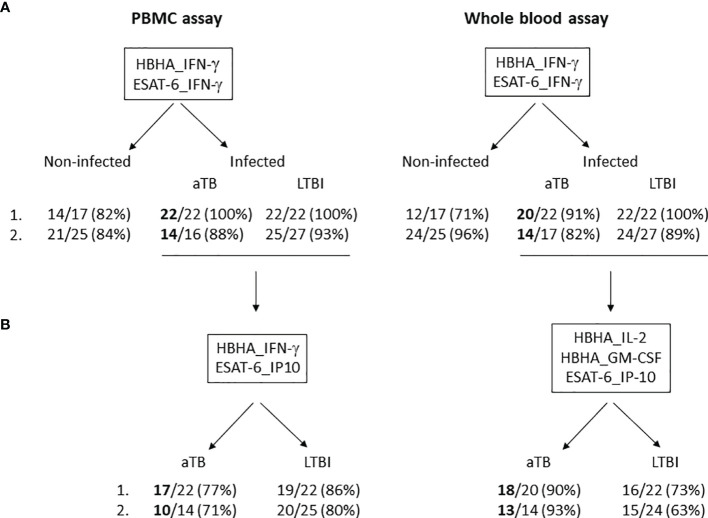
Two-step algorithm for the identification of aTB and LTBI among *M. tuberculosis*-infected subjects by using a combination of cytokines/chemokines induced by two stage-specific mycobacterial antigens in a PBMC- and WB-based assays. **(A)** *M. tuberculosis*-infected subjects, including aTB patients as well as LTBI subjects, were discriminated from non-infected subjects by using a combination of HBHA-IFN-γ and ESAT-6-IFN-γ in a PBMC- (left panel) and a WB-based assay (right panel) in a training (1.) and a validation cohort (2.). The numbers of well-classified subjects are indicated, as well as their percentages. **(B)** Patients with aTB were discriminated from LTBI subjects by using a combination of HBHA-IFN-γ and ESAT-6-IP-10 in a PBMC-based assay (left panel) or of HBHA-IL2, HBHA-GM-CSF and ESAT-6-IP-10 in a WB-based assay (right panel) in a training (1.) and a validation cohort (2.). Only the *M. tuberculosis*-infected subjects well-classified in **(A)** were taken into account for this discrimination between aTB and LTBI. The numbers of well-classified subjects are indicated, as well as their percentages.

These markers were further evaluated in different combinations by logistic regression analysis for the training cohort. This analysis identified the combination of HBHA- and ESAT-6-induced IFN-γ as optimal to differentiate infected from non-infected subjects with 95% correct classification of the individuals (**Figure 1A**). Three non-infected subjects were misclassified. This combination was further applied on the validation cohort. In this case, 88% of the subjects were correctly classified with four controls and four infected subjects (two LTBI subjects and two patients with aTB) misclassified (**Figures 1B**, **2A**).

### Selection of PBMC-Produced Markers to Differentiate aTB From LTBI

**Figure 3:**
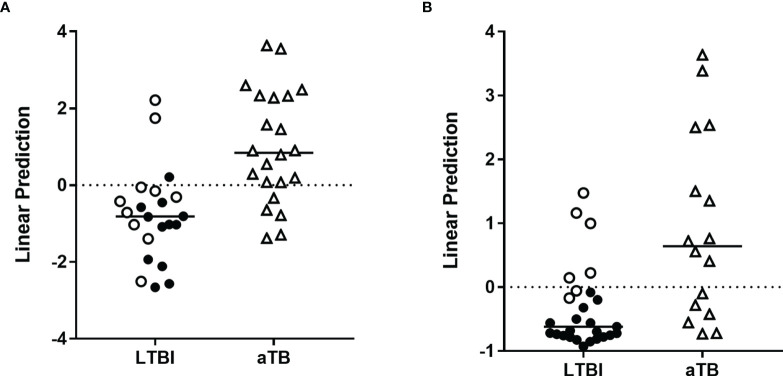
Combination of *M. tuberculosis*-specific immune markers allowing the distinction between LTBI and aTB in the PBMC assay. HBHA-IFN-γ and ESAT-6-IP-10 were the markers selected by logistic regression analysis to be combined for the PBMC-based assay to discriminate aTB from LTBI. Patients with aTB are indicated by open triangles and LTBI subjects at risk to reactivate the infection by open circles. Results are represented with their linear predictor for the validation **(A)** and training **(B)** cohorts. The horizontal lines represent the medians and the dotted lines arbitrary cut-offs.

A second random forest analysis including the same markers was performed to select the optimal combination to differentiate aTB from LTBI in the training cohort. ESAT-6-induced IP-10, IL-8, and HBHA-induced IP-10, IFN-γ, and TNF-α were selected (**Supplementary Figure 1B**) and combinations of these cytokines/chemokines were evaluated by logistic regression analysis. The most discriminant combination was ESAT-6-induced IP-10 combined with HBHA-induced IFN-γ, which provided a correct classification of 82% of the individuals: three LTBI subjects were classified as aTB, whereas five patients with aTB were classified as LTBI (**Figure 3A**). The clinical characteristics of the five aTB patients who were misclassified are reported in **Table 2** (n°1 to n°5). The accuracy of this combination was further evaluated on the validation cohort. As in the validation cohort, two aTB patients and two LTBI subjects were misclassified by the first combination of markers (see **Figure 1B**), all LTBI and aTB individuals were included in this analysis. Seventy-four percent of the infected subjects were correctly classified with five LTBI subjects classified as aTB and six patients with aTB classified as LTBI (**Figure 3B**). The clinical characteristics of the misclassified aTB are reported in **Table 3** (n°1 to 6). Two of them were already missed with the initial combination aiming to discard non-infected controls (n°1 and 2). The five misclassified LTBI subjects were considered at risk to reactivate the infection based on previously defined criteria (21) (**Figure 3B**, open circle).

**Table 2:** Demographic and clinical data of the misclassified aTB patients from the training cohort.

Patient number	Tuberculosis type	Age (years)	Sex	Country of origin	Time since arrival in Belgium	TB risk factors	Sputum smear	Mtb culture	Chest Xray
1	P	30	F	Morocco	7 years	Travels in endemic countries	Positive	Positive	Cavitation
2	P	33	M	Belgium	1 year	Prisoner in high-endemic country, Past-TB	Unknown	Positive	Suspected infiltrates
3	EP lymphadenitis	36	F	Morocco	Unknown	Contact with TB index case	Negative	Negative	Normal
4	P	64	M	Romania	6 months	Contact with TB index case, illegal, alcoholism, tobacco	Positive	Positive	Cavitation
5	EP meningitis	19	M	Morocco	Unknown	Unknown	Negative	Negative	Normal
6	EP spondylodiscitis	30	F	Ivory Coast	5 years	Pregnancy	Negative	Positive	Normal
7	EP Pleural	24	F	Italia-Morocco	4 years	Multiple sclerosis, diabetes, immunosuppressive treatment, contact with TB index case, travels to Marocco	Negative	Positive	Pleural effusion

Mtb, M. tuberculosis; P, pulmonary; EP, extrapulmonary.

**Table 3:** Demographic and clinical data of the misclassified aTB patients from the validation cohort.

Patient number	Tuberculosis type	Age (years)	Sex	Country of origin	Time since arrival in Belgium	TB risk factors	Sputum smear	Mtb culture	Chest Xray
1	P	52	M	Pakistan	Unknown	Diabetes, homeless	Positive	Positive	Cavitations
2	P	35	M	Rwanda	22 years	Homeless, alcoholism, tobacco, denutrition	Positive	Positive*	Cavitation and infiltrates
3	EP lymphadenitis	36	F	Morocco	Unknown	Past-LTBI (1994)	unknown	Positive	Normal
4	P	43	M	Morocco	Unknown	Travels in endemic countries	Positive	Positive	Cavitation and infiltrates
5	P	63	M	Morocco	Unknown	Travels in endemic countries	Positive	Positive*	Cavitation
6	P	45	M	Poland	Unknown	Homeless, Alcoholic hepatitis/Acute cirrhosis	unknown	Positive	Bilateral nodules
7	P	45	M	Romania	1 year and 7 months	Alcoholism	Positive	Positive*	Infiltrate
8	P	46	M	Cameroun	Unknown	Unknown	unknown	Positive	Pulmonary condensation, mediastinal adenopathies

Mtb, M. tuberculosis; P, pulmonary; EP, extrapulmonary; * positive polymerase chain reaction.

A good performance of the differential diagnosis between aTB and LTBI was thus possible in most cases by combining ESAT-6-induced IP-10 to HBHA-induced IFN-γ. However, this combination did not allow us to identify the two aTB patients who were initially misclassified as non-infected controls. The two-steps approach with a first identification of infected subjects, followed by a differential diagnosis between aTB and LTBI among infected individuals remains thus recommended as illustrated in **Figure 2**, panel A and B respectively. This two-steps approach resulted for the two cohorts in a correct identification of 36/38 aTB patients as infected (**Figure 2A**), with 27 of them as aTB patients (**Figure 2B**).

### Selection of WB Markers to Identify *M. tuberculosis-*Infected Subjects

The same approach was applied for the WB assays. Similar to the PBMC, cytokine concentrations were generally significantly higher in infected than in non-infected individuals. ROC curves analyses differentiated infected from non-infected subjects with AUCs generally higher than 0.7 (**Supplementary Table 2**). Random forest analyses of the 13 analytes secreted in response to ESAT-6 and HBHA classified HBHA-induced IL-2, TNF-α and IFN-γ, together with ESAT-6-induced IL-8, TNF-α, IP-10 and IFN-γ as the optimal markers (**Supplementary Figure 1C**).

**Figure 4:**
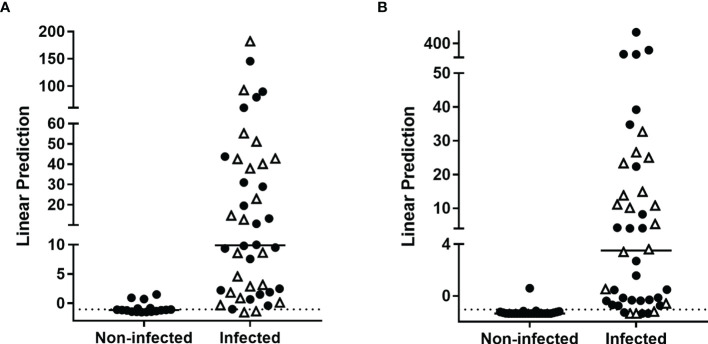
Combination of *M. tuberculosis*-specific immune markers allowing the distinction between *M. tuberculosis*-infected versus non-infected subjects in the WB assay. HBHA-IFN-γ and ESAT-6-IFN-γ were the markers selected by logistic regression analysis to be combined for the WB-based assay to discriminate *M. tuberculosis*-infected from non-infected subjects. Patients with aTB are indicated by open triangles. Results are represented with their linear predictor for the validation **(A)** and training **(B)** cohorts. The horizontal lines represent the medians and the dotted lines arbitrary cut-offs.

Logistic regression analysis on the results obtained for the training cohort identified three promising combinations of markers to be used to rule-out infection: HBHA- and ESAT-6-induced IFN-γ, HBHA- and ESAT-6-induced TNF-α, HBHA-induced IL-2 and ESAT-6-induced TNF-α. The first combination provided the best results on both cohorts. Eighty-nine percent and 90% of the subjects were correctly classified as infected or not in the training and validation cohorts, respectively (**Figures 4A, B**). Five controls and two aTB were misclassified in the training cohort as well as one control, three LTBI and three aTB in the validation cohort (**Figures 2A**, **3A, B**). Three misclassified controls and two misclassified aTB patients with the WB assay were also misclassified in the PBMC assay. The clinical characteristics of the misclassified aTB patients are provided in **Table 2** (n°5 and n°6) for the training cohort and in **Table 3** (n°1, n°2 and n°7) for the validation cohort.

### Selection of WB Markers to Differentiate aTB From LTBI

**Figure 5:**
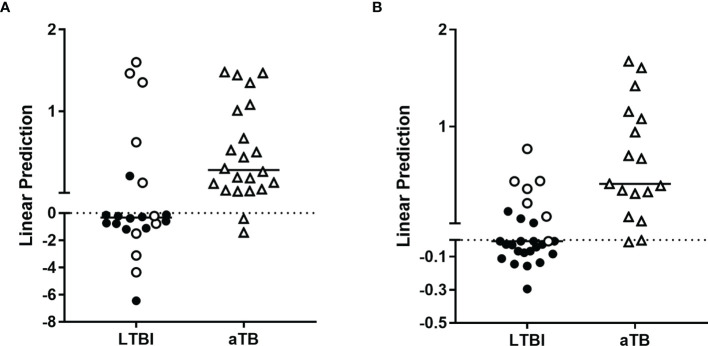
Combination of *M. tuberculosis*-specific immune markers allowing the distinction between LTBI and aTB in the WB assay. HBHA-IL2, HBHA-GM-CSF and ESAT-6-IP-10 were the host markers selected by logistic regression analysis to be combined for the WB-based assay to discriminate aTB from LTBI. Patients with aTB are indicated by open triangles and LTBI subjects at risk to reactivate the infection by open circles. Results are represented with their linear predictor for the validation **(A)** and training **(B)** cohorts. The horizontal lines represent the medians and the dotted lines arbitrary cut-offs.

A second Random forest analysis was performed to identify the best markers to differentiate aTB from LTBI in the training cohort. All infected individuals were included in this analysis as a few patients with aTB were not identified as infected by the previous combination. ESAT-6-induced IP-10, and HBHA-induced IL-8, IL-2 and GM-CSF were identified as the best markers to differentiate aTB from LTBI (**Supplementary Figure 1D**). The optimal combination provided by further logistic regression was ESAT-6-induced IP-10 combined with HBHA-induced IL-2 and GM-CSF, which allowed us to correctly classify 82% of the subjects (**Figure 5A**). Among the misclassified subjects, only two were aTB patients (**Table 2**, n°4 and n°7), while the other six were LTBI subjects.

In the validation cohort, this combination correctly classified 75% of the subjects (**Figure 5B**). As for the training cohort, only two aTB patients were misclassified (**Table 3**, n°1 and n°8), whereas the other nine misclassified subjects were LTBI subjects.

Good differential diagnosis between aTB and LTBI was thus possible in most cases by combining ESAT-6-induced IP-10 to HBHA-induced IL-2 and GM-CSF. However, this combination did not allow us to identify all the aTB patients who were initially misclassified as non-infected controls. Applying the two-steps approach to first identify infected subjects and then to identify aTB patients among infected individuals resulted globally for the two cohorts in a correct identification of 31/34 aTB patients.

This combination misclassified several LTBI subjects but most of them (11/15) were considered as being at risk to reactivate their infection.

## Discussion

TB control could be significantly improved if simple and rapid triage or rule-out tests were available to first exclude non-infected subjects and then differentiate as much as possible aTB from LTBI. The development of triage tests designed for use by first-contact health care providers as a rule-out test of TB was reported by the World Health Organization as a high priority need for TB control (22). This strategy would limit the number of individuals requiring a confirmatory test. High sensitivity is needed for this approach to avoid missing patients with aTB. In contrast, the specificity of this triage test approach may be lower, as the patients initially selected by the triage test will benefit from more in-depth evaluation of their status. Blood-based immunological tests might be appropriate as triage tests. We previously reported that secretion of IFN-γ by PBMC or WB in response to *in vitro* stimulation with HBHA is a biomarker for LTBI contrasting with ESAT-6-induced IFN-γ secretion that is more characteristic of aTB (14, 15). Therefore, we suggested that combining IFN-γ secretion in response to HBHA and to ESAT-6 would help to differentiate LTBI from aTB and allows to stratify LTBI subjects in different groups associated with risks of reactivation of the infection (16, 21). In this study, we evaluated combinations of cytokines/chemokines released in response to these two antigens for improved identification of aTB patients, which could then be proposed as a triage test to select patients likely presenting aTB for further investigations to confirm the diagnosis. We propose a two-step approach consisting of first identifying infected subjects and then differentiating aTB from LTBI among infected individuals. Aiming to provide an easy test, we searched for optimal cytokine/chemokine combinations secreted by 24 hours-stimulated WB. However, this approach might not be sensitive enough for patients with lymphopenia or important inflammatory syndrome associated with high levels of plasma proteins that could inhibit cellular immune responses. It might also not be suitable for frozen material. We therefore also evaluated optimal cytokine/chemokine combinations secreted after 24 hours of *in vitro*-stimulated PBMC.

Among the 13 measured cytokines/chemokines secreted in response to HBHA and ESAT-6 and their various combinations to identify *M. tuberculosis*-infected patients, the best results were obtained both on WB and on PBMC by combining IFN-γ concentrations secreted in response to HBHA and to ESAT-6, as previously shown (15, 16). This approach allowed us to discard for further analysis most non-infected controls, 82%/71% in the training cohort and 84%/96% in the validation cohort, in the PBMC and WB assays, respectively. Misclassified controls were most often doubtful rather than being clearly positive. In addition, among the 10 misclassified controls in the PBMC and/or WB assay for both cohorts, five were health care workers potentially exposed to aTB patients, and four reported frequent travel to high TB endemic countries. Although these individuals were classified as non-infected, based on a negative TST result as recommended in Belgium (23), we cannot formally exclude that they developed immune responses to mycobacterial antigens as a consequence of exposure to *M tuberculosis*. They represented a minority of the enrolled controls that should benefit from further investigation (medical visit, chest radiograph, eventually followed by microbiological analysis of sputum samples) to exclude an ongoing *M. tuberculosis* infection. On the other hand, most infected individuals and especially most aTB patients were correctly identified and were selected for further investigations (100%/91% and 88%/82% in the training cohort in the validation cohort, in the PBMC and WB assays, respectively). Among the five misclassified aTB patients either in the PBMC or the WB assay in both cohorts, two had extra-pulmonary TB (one meningitis and one spondylodiscitis in a pregnant women) with obvious symptoms, while the other three had symptomatic pulmonary TB with severe denutrition and serious abnormalities on chest radiographs (**Tables 2** and **3**).

As a second step, we searched for the best combination of cytokines/chemokines secreted by the two stage-specific mycobacterial antigens to allow us to identify patients with aTB among infected individuals. Results were slightly different between the PBMC and WB assays. For PBMC stimulation, the best combination was HBHA-induced IFN-γ and ESAT-6-induced IP-10 that allowed us to correctly identify 77% and 71% of aTB patients in the training and validation cohorts, respectively. The interest of ESAT-6-induced IP-10 for diagnosis of *M. tuberculosis* infection was previously reported but with little added value compared to IFN-γ, except for patients with immune deficiencies (24). ESAT-6-induced IP-10 was not reported to provide differential diagnosis between aTB and LTBI. However, we show here that, when combined with HBHA-induced IFN-γ, it improves the differentiation between aTB and LTBI. This differentiation allowed us to identify ¾ of the patients with aTB, which is an improvement over previous studies. In addition, most misclassified aTB patients were shown to be infected in the first stage analysis and will therefore receive clinical attention.

Globally, eight LTBI subjects were misclassified as aTB. Seven of them were considered in a previous study as being at risk to reactivate their infection, based on their high ESAT-6-induced IFN-γ secretion (21). Latency is considered as a spectrum of different stages of activity of persistent mycobacteria, from totally quiescent to persistently multiplying (17, 18). This latter stage is the highest risk stage and therefore may not be possible to be differentiated from subclinical TB using immunological biomarkers. We may therefore hypothesize that these 8/49 misclassified LTBI subjects might have subclinical TB. Therefore, these individuals should be prioritized for more extensive evaluation for a possible aTB.

For WB stimulation, the best discrimination between aTB and LTBI was obtained by combining HBHA-induced IL-2 and GM-CSF with ESAT-6-induced IP-10. This allowed us to identify 90% and 93% of the aTB patients among infected individuals from the training and validation cohorts, respectively. The main advantages of the WB assay are the relatively small blood volume required (feasible on 1 mL WB) and easiness to perform. The good sensitivity should allow us to propose these tests as a triage test for aTB as the recommended sensitivity is to be at least 90% (22), and should help to limit the number of individuals who require confirmatory tests. One third of the LTBI subjects were misclassified and would require the confirmatory test. However, as for LTBI subjects misclassified with the PBMC assay, most of them were considered at risk to reactivate the infection and should therefore be prioritized for further evaluation of their status.

The sensitivities achieved with these combinations are similar to those reported in a few other studies aiming to define combinations of biomarkers that provide diagnostic test accuracy consistent with WHO specifications for a rule-out test for aTB. These studies that were recently summarized (25), are based on combinations of various serum markers, mostly cytokines/chemokines (26–28), sometimes combined with antibodies against a TB antigen to raise the specificity for TB (29). We chose in our study to measure cytokines/chemokines released in response to mycobacterial antigens to increase the specificity for TB. A few studies already addressed this question by measuring chemokines/cytokines induced by the peptides from the QuantiFERON, with different marker selections among the studies (13). Our results are difficult to compare to these studies, as we selected combinations of chemokines/cytokines induced by two different stage-specific mycobacterial antigens, ESAT-6 and HBHA, in order to cover a wide range of the *M. tuberculosis* metabolism. ESAT-6 is highly expressed during bacterial multiplication, while HBHA is a latency-associated antigen, whose gene is upregulated in hypoxic conditions and in cells harboring *M. tuberculosis* during latency (30–32). Not surprisingly, the selected combinations to differentiate aTB from LTBI were different between the PBMC and WB assays, since during acute inflammation plasma proteins may modify cellular immune responses.

Strengths of our study are that the biomarkers identified in the training cohort were validated in an independent validation cohort, both for the PBMC and the WB assays, and that these biomarkers are easy to measure in most laboratories. In addition, as blood and not sputum-based biomarkers, they are suitable to identify both pulmonary and extra-pulmonary TB who were both included in our cohorts. A limitation of this study results from the relatively low numbers of individuals in each cohort and from the heterogeneity of the cohorts that comprised both patients with pulmonary and extra-pulmonary TB. Moreover, we did not include in our cohorts TB-like diseases, which may perhaps represent more relevant negative controls for the evaluation of biomarkers to diagnose aTB. Inclusion of TB-like diseases was unfortunately not possible, because no screening for LTBI is performed in these patients in Belgium so that correct classification of these patients would have been difficult.

Based on the results of this pilot study performed in a low TB incidence country, we propose a two-steps algorithm to identify patients who are highly suspected to present aTB and should be further investigated (**Figure 2**). This algorithm warrants further evaluation on larger cohorts of subjects both in low and high TB endemic countries to confirm its robustness as triage test for aTB. Albeit not as simple as recommended to be a point of care test (22), it has the advantage of being relatively easy to perform on small blood volumes, to provide acceptable identification of aTB patients, and to be less expensive than systems biology approaches that have identified diagnostic signatures to discriminate aTB from LTBI (33, 34).

## Data Availability Statement

The raw data supporting the conclusions of this article will be made available by the authors, without undue reservation.

## Ethics Statement

The studies involving human participants were reviewed and approved by Ethics Committee of ULB-Hôpital Erasme, Brussels, Belgium. The patients/participants provided their written informed consent to participate in this study.

## Author Contributions

VD designed the study, analyzed the data and wrote a first draft of the manuscript. PC performed the complete statistical analysis of the data. AP performed all the experimental work. MH, ND, and SA included patients, provided clinical data and reviewed the manuscript. JR did the initial statistical analysis. MS prepared ESAT-6 antigens. CL coordinated the preparation of antigens and critically reviewed the manuscript. FM designed the study, interpreted the data, and drafted the final manuscript. VC designed the study, analyzed and interpreted the data, collected clinical data, drafted figures and tables and critically reviewed the manuscript. All the authors contributed to this article and approved the submitted version.

## Funding

This work was supported by the Fonds National de la Recherche Scientifique (FNRS—PDR T.0147.13), by the European Community within the Seventh Framework Program (FP7) NEWTBVAC (grant HEALTH-2009-2.3.2-2), and within the Horizon2020 program TBVAC2020 (grant agreement 643381), and by Innoviris Brussels.

## Conflict of Interest

The author MS is employed by Lionex Diagnostics and Therapeutics, Braunschweig, Germany.

The remaining authors declare that the research was conducted in the absence of any commercial or financial relationships that could be construed as a potential conflict of interest.

## Publisher’s Note

All claims expressed in this article are solely those of the authors and do not necessarily represent those of their affiliated organizations, or those of the publisher, the editors and the reviewers. Any product that may be evaluated in this article, or claim that may be made by its manufacturer, is not guaranteed or endorsed by the publisher.

## References

[1] World Health Organization. WHO Global Tuberculosis Report (2021). Available at: http://www.who.int/tb/publications/global_report/en/ (Accessed November 1, 2021).

[2] Drain PK Bajema KL Dowdy D Dheda K Naidoo K Schumacher SG. Incipient and Subclinical Tuberculosis: A Clinical Review of Early Stages and Progression of Infection. Clin Microbiol Rev (2018) 31(4):e00021–18. doi: 10.1128/CMR.00021-18 PMC6148193 30021818

[3] Dinnes J Deeks J Kunst H Gibson A Cummins E. A Systematic Review of Rapid Diagnostic Tests for the Detection of Tuberculosis Infection. Health Technol Assess (2007) 11:1–196. doi: 10.3310/hta11030 17266837

[4] De Visser V Sotgiu G Lange C Aabye MG Bakker M Bartalesi F. False-Negative Interferon-γ Release Assay Results in Active Tuberculosis: A TBNET Study. Eur Respir J (2015) 45:279–83. doi: 10.1183/09031936.00120214 25359336

[5] Santos JA Duarte R Nunes C. Host Factors Associated to False Negative and Indeterminate Results in an Interferon-γ Release Assay in Patients With Active Tuberculosis. Pulmonology (2019) 26(6):353–62. doi: 10.1016/j.pulmoe.2019.11.001 31843341

[6] Sester M Sotgiu G Lange C Giehl C Girardi E Migliori GB. Interferon-γ Release Assays for the Diagnosis of Active Tuberculosis: A Systematic Review and Meta-Analysis. Eur Respir J (2011) 37:100 –111. doi: 10.1183/09031936.00114810 20847080

[7] Lange C Pai M Drobniewsli F Migliori GB. Interferon-γ Release Assays for the Diagnosis of Active Tuberculosis: Sensible or Silly? Eur Respir J (2009) 33:1250–3. doi: 10.1183/09031936.00019709 19483044

[8] Shafeque A Bigio J Hogan CA Pai M Banaei N. Fourth-Generation QuantiFERON-TB Gold Plus: What Is the Evidence? J Clin Microbiol (2020) 58(9):e01950–19. doi: 10.1128/JCM.01950-19 PMC7448650 32493779

[9] Della Bella C Spinicci M Ainwaisri HFM Bartalesi F Tapinassi S Mencarini J. LIOFeron^®^TB/LTBI: A Novel and Reliable Test for LTBI and Tuberculosis. J Infect Dis (2020) 91:177–81. doi: 10.1016/j.ijid.2019.12.012 31877486

[10] Molicotti P Bua A Cubeddu M Cannas S Delogu G Zanetti S. Tuberculosis Patients Are Characterized by a Low-IFN-γ/High-TNF-α Response to Methylated HBHA Produced in M. Smegmatis. Diagn Microbiol Infect Dis (2011) 71(4):449–52. doi: 10.1016/j.diagmicrobio.2011.08.010 22083081

[11] Loxton AG Black GF Stanley K Walzl G. HBHA Induces IFN-γ+ IL-2+ IL-17+ T Cells During Multifunctional CD4+ T Cells During Latent But Not Active Tuberculosis Disease. Clin Vaccine Immunol (2012) 19(5):746–51. doi: 10.1128/CVI.00047-12 PMC3346316 22461525

[12] Dreesman A Corbière V Dirix V Smits K Debulpaep S De Schutter I. Age-Stratified T Cell Responses in Children Infected With Mycobacterium Tuberculosis. Front Immunol (2017) 8:1059. doi: 10.3389/fimmu.2017.01059 28928738 PMC5591888

[13] Qiu B Liu Q Li Z Song H Xu D Ji Y. Evaluation of Cytokines as a Biomarker to Distinguish Active Tuberculosis From Latent Tuberculosis Infection: A Diagnostic Meta-Analysis. BMJ Open (2020) 10:e039501. doi: 10.1136/bmjopen-2020-039501 PMC7542925 33033030

[14] Hougardy JM Schepers K Place S Drowart A Lechevin V Verscheure V. Heparin-Binding-Hemagglutinin-Induced IFN-γ Release as a New Diagnostic Tool for Latent Tuberculosis. PloS One (2007) 10:e926. doi: 10.1371/journal.pone.0000926 PMC1991599 17912342

[15] Mascart F Locht C. Integrating Knowledge of *Mycobacterium Tuberculosis* Pathogenesis for the Design of Better Vaccines. Expert Rev Vaccines (2015) 14(12):1573–85. doi: 10.1586/14760584.2015.1102638 26517361

[16] Dirix V Dauby N Hites M Watelet E Van Praet A Godefroid A. Optimal Detection of Latent M. Tuberculosis Infection by Combined HBHA and ESAT-6-Whole Blood Interferon-γ-Release Assays. J Clin Microbiol (2022) in press. 10.1128/jcm.02443-21 PMC9116186 35430897

[17] Lin PL Flynn JL. The End of the Binary Era: Revisiting the Spectrum of Tuberculosis. J Immunol (2018) 201(9):2541–8. doi: 10.4049/jimmunol.1800993 PMC6217958 30348659

[18] Boom WH Schaible UE Achkar JM. The Knows and Unknows of Latent Mycobacterium Tuberculosis Infection. J Clin Invest (2021) 131(3):e136222. doi: 10.1172/JCI136222 PMC7843221 33529162

[19] Wyndham-Thomas C Corbière V Dirix V Smits K Domont F Libin M. Key Role of Effector Memory CD4^+^ T Lymphocytes in a Short-Incubation Heparin-Binding Hemagglutinin Gamma Interferon Release Assay for Detection of Latent Tuberculosis. Clin Vaccine Immunol (2014) 21(3):321–8. doi: 10.1128/CVI.00651-13 PMC3957667 24391135

[20] Corbière V Segers J Desmet R Lecher S Loyens M Petit E. Natural T Cell Epitope Containing Methyl Lysines on Mycobacterial Heparin-Binding Hemagglutinin. J Immunol (2020) 204:1715–23. doi: 10.4049/jimmunol.1901214 32122997

[21] Corbière V Pottier G Bonkain F Schepers K Verscheure V Lecher S. Risk Stratification of Latent Tuberculosis Defined by Combined Interferon Gamma Release Assays. PloS One (2012) 7(8):e43285. doi: 10.1371/journal.pone.0043285 22912846 PMC3422279

[22] Denkinger CM Kik SV Cirillo DM Casenghi M Shinnick T Weyer K. Defining the Needs for Next Generation Assays for Tuberculosis. J Infect Dis (2015) 211(Suppl 2):S29–38. doi: 10.1093/infdis/jiu821 PMC4447829 25765104

[23] Fonds Des Affections Respiratoires. Tuberculose. Available at: http://www.fares.be (Accessed December 15th 2021).

[24] Ruhwald M Aabye MG Ravn P. IP-10 Release Assays in the Diagnosis of Tuberculosis Infection: Current Status Ad Future Directions. Expert Rev Mol Diagn (2012) 12(2):175–87. doi: 10.1586/erm.11.97 22369377

[25] Escalante P Theel ES. Progress Towards Developing a Rapid Triage/Referral Test for Tuberculosis. Clin Chem (2020) 66(8):995–7. doi: 10.1093/clinchem/hvaa1 32642751

[26] Chegou NN Sutherland JS Malherbe S Crampin AC Corstjens PLAM Geluk A. Diagnostic Performance of a Seven-Marker Serum Protein Biosignature for the Diagnosis of Active TB Disease in African Primary Healthcare Clinic Attendees With Signs and Symptoms Suggestive of TB. Thorax (2016) 71(9):785–94. doi: 10.1136/thoraxjnl-2015-20799925 27146200

[27] De Groote MA Sterling DG Hraha T Russell TM Green LS Wall K. Discovery and Validation of Six-Marker Serum Protein Signature for the Diagnosis of Active Pulmonary Tuberculosis. J Clin Microbiol (2017) 55(10):3057–71. doi: 10.1128/JCM.00467-17 PMC5625392 28794177

[28] Delemarre EM van Hoorn L Bossink AWJ Drylewicz J Joosten SA Ottenhoff THM. Serum Biomarker Profile Including CCL1, CXCL10, VEGF, and Adenosine Deaminase Activity Distinguishes Active From Remotely Acquired Latent Tuberculosis. Front Immunol (2021) 12:725447. doi: 10.3389/fimmu.2021.725447 34691031 PMC8529994

[29] Ahmad R Xie L Pyle M Suarez MF Broger T Steinberg D. A Rapid Triage Test for Active Pulmonary Tuberculosis in Adult Patients With Persistent Cough. Sci Transl Med (2019) 11(515):eaaw8287. doi: 10.1126/scitranslmed.aaw8287 31645455

[30] Mack U Migliori GB Sester M Rieder HL Ehlers S Goletti D. LTBI: Latent Tuberculosis Infection or Lasting Immune Responses to *M. Tuberculosis*? A TBNET Consensus Statement. Eur Respir J (2009) 33:956–73. doi: 10.1183/09031936.00120908 19407047

[31] Iona E Pardini M Mustazzolu A Piccaro G Nisini R Fattorini L. Mycobacterium Tuberculosis Gene Expression at Different Stages of Hypoxia-Induced Dormancy and Upon Resuscitation. J Microbiol (2016) 54:565–72. doi: 10.1007/s12275-016-6150-4 27480637

[32] Delogu G Sanguinetti M Posteraro B Rocca S Zanetti S Fadda G. The hbhA Gene of *Mycobacterium Tuberculosis* Is Specifically Upregulated in the Lungs But Not in the Spleens of Aerogenically Infected Mice. Infect Immun (2006) 74:3006–11. doi: 10.1128/IAI.74.5.3006-3011.2006 PMC1459695 16622240

[33] Bloom CI Graham CM Berry MP Rozakeas F Redford PS Wang Y. Transcriptional Blood Signatures Distinguish Pulmonary Tuberculosis, Pulmonary Sarcoidosis, Pneumonias and Lung Cancers. PloS One (2013) 8(8):e70630. doi: 10.1371/journal.pone.0070630 23940611 PMC3734176

[34] Kaforou M Wright VJ Oni T French N Anderson ST Bangani N. Detection of Tuberculosis in HIV-Infected and -Uninfected African Adults Using Whole Blood RNA Expression Signatures: A Case-Control Study. PloS Med (2013) 10(10):e1001538. doi: 10.1371/journal.pmed.1001538 24167453 PMC3805485

