# Supplementary material for: Immuno-Diagnosis of Active Tuberculosis by a Combination of Cytokines/Chemokines Induced by Two Stage-Specific Mycobacterial Antigens: A Pilot Study in a Low TB Incidence Country
Source: Front Immunol. 2022 Mar 10;13:842604. doi: 10.3389/fimmu.2022.842604 (PMC8960450; doi:10.3389/fimmu.2022.842604)
Supplement: Supplementary file 1 [file Image_1.pdf]

Supplementary Figure 1

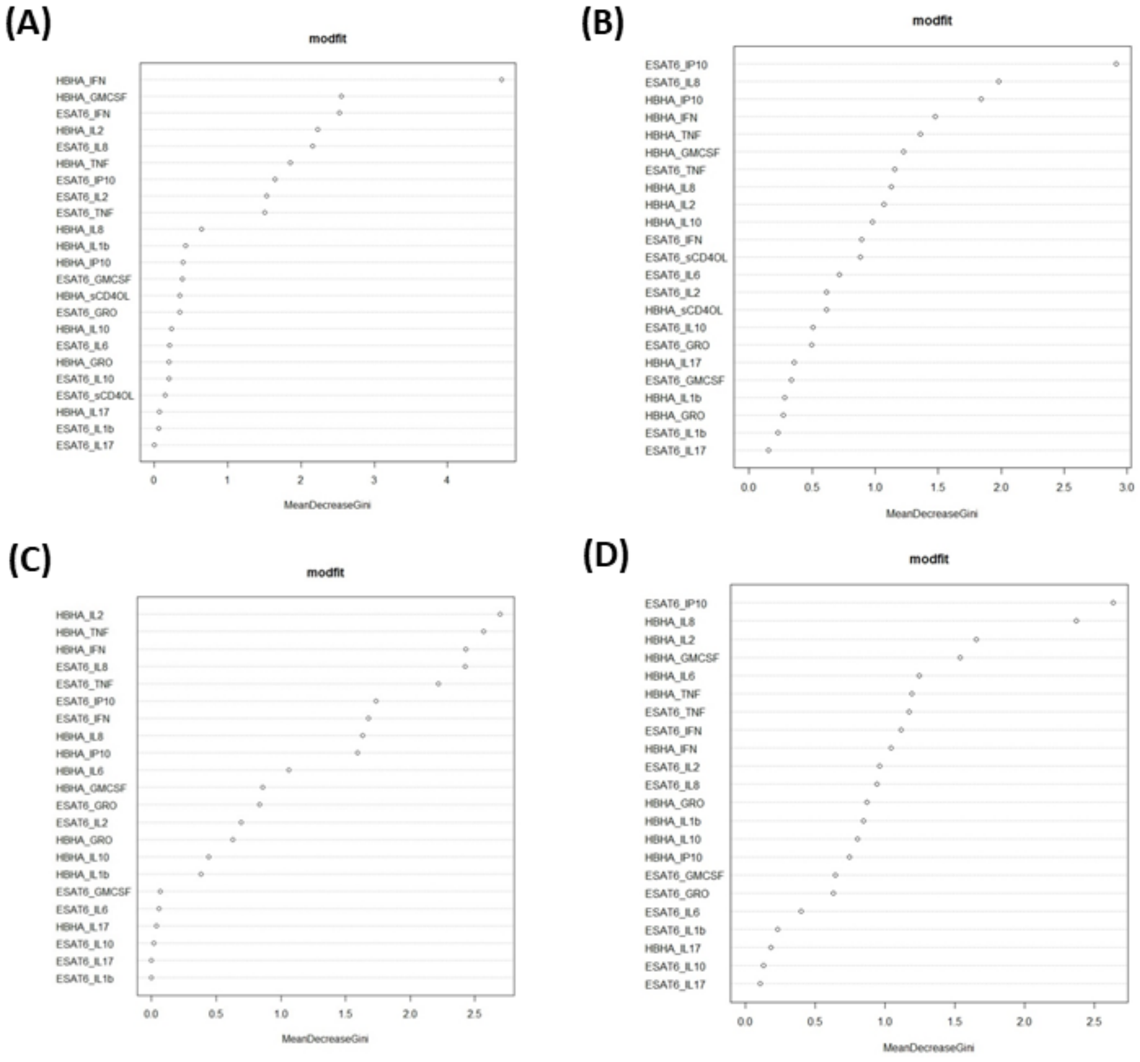

Supplementary Figure 1. Random forest analysis classifying the best markers to discriminate *M. tuberculosis*-infected adults from the non-infected controls. Prediction of the best individual markers (from the top to the bottom of the list) correctly classifying the *M. tuberculosis*-infected and non-infected subjects (A, C) or aTB patients from LTBI subjects (B, D) by a random forest analysis for the PBMC- (A, B) and WB- (C, D) based assays.
