# Supplementary material for: Immuno-Diagnosis of Active Tuberculosis by a Combination of Cytokines/Chemokines Induced by Two Stage-Specific Mycobacterial Antigens: A Pilot Study in a Low TB Incidence Country
Source: Front Immunol. 2022 Mar 10;13:842604. doi: 10.3389/fimmu.2022.842604 (PMC8960450; doi:10.3389/fimmu.2022.842604)
Supplement: Supplementary file 2 [file Table_1.docx]

**SUPPLEMENTARY MATERIAL**

**SUPPLEMENTARY TABLES**

**Supplementary Table 1.** HBHA- and ESAT-6-induced cytokines in PBMC-supernatants of infected and non-infected subjects from the training and validation cohort

|  | **Training cohort** | | | | | | | | **Validation cohort** | | | | | | | |
| --- | --- | --- | --- | --- | --- | --- | --- | --- | --- | --- | --- | --- | --- | --- | --- | --- |
| **Host-marker** | **HBHA** | | | | **ESAT-6** | | | | **HBHA** | | | | **ESAT-6** | | | |
|  | **Median [P25-P75]** | | **p*** | **Area under ROC curve** | **Median [P25-P75]** | | **p*** | **Area under ROC curve** | **Median [P25-P75]** | | **p*** | **Area under ROC curve** | **Median [P25-P75]** | | **p*** | **Area under ROC curve** |
|  | **Non-infected** | **Infected** |  |  | **Non-infected** | **Infected** |  |  | **Non-infected** | **Infected** |  |  | **Non-infected** | **Infected** |  |  |
| **GM-CSF** | 5 | 44 | <0.0001 | 0.892 | 5 | 17 | <0.0001 | 0.796 |  |  |  |  |  |  |  |  |
|  | [5-5] | [14-139] |  |  | [5-5] | [5-50] |  |  |  |  |  |  |  |  |  |  |
| **Gro** | 2500 | 2500 | 0.843 | 0.515 | 248 | 1053 | 0.1409 | 0.619 |  |  |  |  |  |  |  |  |
|  | [103-2500] | [341-2500] |  |  | [141-596] | [5-2500] |  |  |  |  |  |  |  |  |  |  |
| **IFN-γ** | 31 | 991 | <0.0001 | 0.967 | 5 | 160 | <0.0001 | 0.880 | 10 | 149 | <0.0001 | 0.889 | 10 | 47 | <0.0001 | 0.774 |
|  | [18-51] | [227-3607] |  |  | [5-5] | [22-1037] |  |  | [10-21] | [31-599] |  |  | [10-10] | [10-455] |  |  |
| **IL-1β** | 5 | 16 | 0.0002 | 0.773 | 5 | 5 | 0.0056 | 0.682 |  |  |  |  |  |  |  |  |
|  | [5-5] | [5-171] |  |  | [5-5] | [5-18] |  |  |  |  |  |  |  |  |  |  |
| **IL-2** | 14 | 132 | <0.0001 | 0.887 | 5 | 52 | <0.0001 | 0.860 |  |  |  |  |  |  |  |  |
|  | [10-27] | [37-201] |  |  | [5-5] | [5-164] |  |  |  |  |  |  |  |  |  |  |
| **IL-6** | 5 | 85 | 0.015 | 0.693 | 5 | 5 | 0.0057 | 0.682 |  |  |  |  |  |  |  |  |
|  | [5-162] | [5-1251] |  |  | [5-5] | [5-158] |  |  |  |  |  |  |  |  |  |  |
| **IL-8** | 4145 | 35058 |  |  | 1499 | 13380 | <0.0001 | 0.856 |  |  |  |  |  |  |  |  |
|  | [2152-26317] | [7983-84523] | 0.0008 | 0.771 | [345-2316] | [3478-44109] |  |  |  |  |  |  |  |  |  |  |
| **IL-10** | 15 | 42 | 0.0474 | 0.664 | 5 | 7 | 0.0330 | 0.666 |  |  |  |  |  |  |  |  |
|  | [8-60] | [15-138] |  |  | [5-9] | [5-20] |  |  |  |  |  |  |  |  |  |  |
| **IL-17A** | 5 | 5 | 0.0774 | 0.602 | 5 | 5 | 0.1645 | 0.580 |  |  |  |  |  |  |  |  |
|  | [5-5] | [5-6] |  |  | [5-5] | [5-5] |  |  |  |  |  |  |  |  |  |  |
| **IP-10** | 9000 | 18212 | 0.0904 | 0.641 | 780 | 13729 | <0.0001 | 0.829 |  |  |  |  | 489 | 10086 | 0.0001 | 0.771 |
|  | [4334-20962] | [7734-41049] |  |  | [5-3231] | [4427-49644] |  |  |  |  |  |  | [10-3544] | [1706-34680] |  |  |
| **MIP-1α** | 25 | 257 | 0.0003 | 0.808 | 12 | 130 | 0.0019 | 0.768 |  |  |  |  |  |  |  |  |
|  | [5-915] | [46-2692] |  |  | [7-22] | [11-701] |  |  |  |  |  |  |  |  |  |  |
| **sCD40L** | 5 | 20 | 0.0007 | 0.764 | 5 | 9 | 0.0024 | 0.729 |  |  |  |  |  |  |  |  |
|  | [5-7] | [5-98] |  |  | [5-5] | [5-23] |  |  |  |  |  |  |  |  |  |  |
| **TNF-α** | 34 | 325 | <0.0001 | 0.866 | 5 | 116 | <0.0001 | 0.876 |  |  |  |  |  |  |  |  |
|  | [18-118] | [114-925] |  |  | [5-15] | [28-242] |  |  |  |  |  |  |  |  |  |  |

Results of the measured concentrations are reported in pg/ml as medians and 25^th^ – 75^th^ percentiles. The degrees of significance of the differences between the concentrations measured in the two groups of subjects are reported as *p* values. The diagnostic ability of each cytokine was assessed by receiver operator characteristics (ROC) curve analysis and the areas under the curves are reported in the table. *Mann-Whitney U test

**Supplementary Table 2.** HBHA- and ESAT-6-induced cytokines in WB-supernatants of infected and non-infected subjects from the training and validation cohort

|  | **Training cohort** | | | | | | | | **Validation cohort** | | | | | | | |
| --- | --- | --- | --- | --- | --- | --- | --- | --- | --- | --- | --- | --- | --- | --- | --- | --- |
| **Host-marker** | **HBHA** | | | | **ESAT-6** | | | | **HBHA** | | | | **ESAT-6** | | | |
|  | **Median [P25-P75]** | | **p*** | **Area under ROC curve** | **Median [P25-P75]** | | **p*** | **Area under ROC curve** | **Median [P25-P75]** | | **p*** | **Area under ROC curve** | **Median [P25-P75]** | | **p*** | **Area under ROC curve** |
|  | **Non-infected** | **Infected** |  |  | **Non-infected** | **Infected** |  |  | **Non-infected** | **Infected** |  |  | **Non-infected** | **Infected** |  |  |
| **GM-CSF** | 5 | 91 | <0.0001 | 0.816 | 5 | 5 | 0.0015 | 0.716 | 10 | 17 | <0.0001 | 0.798 |  |  |  |  |
|  | [5-20] | [20-209] |  |  | [5-5] | [5-46] |  |  | [10-10] | [10-40] |  |  |  |  |  |  |
| **Gro** | 1577 | 2500 | 0.0416 | 0.655 | 29 | 560 | 0.0005 | 0.7767 |  |  |  |  |  |  |  |  |
|  | [608-2500] | [1295-2500] |  |  | [5-175] | [90-2500] |  |  |  |  |  |  |  |  |  |  |
| **IFN-γ** | 55 | 763 | <0.0001 | 0.845 | 5 | 114 | <0.0001 | 0.851 | 10 | 188 | <0.0001 | 0.827 | 10 | 40 | <0.0001 | 0.762 |
|  | [26-118] | [228-4406] |  |  | [5-11] | [17-411] |  |  | [10-16] | [15-342] |  |  | [10-10] | [10-454] |  |  |
| **IL-1β** | 5 | 17 | 0.0005 | 0.763 | 5 | 5 | 0.0745 | 0.602 |  |  |  |  |  |  |  |  |
|  | [5-5] | [5-91] |  |  | [5-5] | [5-5] |  |  |  |  |  |  |  |  |  |  |
| **IL-2** | 24 | 244 | <0.0001 | 0.850 | 5 | 32 | 0.0001 | 0.782 | 10 | 28 | <0.0001 | 0.818 |  |  |  |  |
|  | [15-55] | [80-471] |  |  | [5-5] | [5-144] |  |  | [10-10] | [10-59] |  |  |  |  |  |  |
| **IL-6** | 5 | 394 | <0.0001 | 0.810 | 5 | 5 | 0.0125 | 0.659 |  |  |  |  |  |  |  |  |
|  | [5-74] | [60-3024] |  |  | [5-5] | [5-267] |  |  |  |  |  |  |  |  |  |  |
| **IL-8** | 5731 | 26189 | <0.0001 | 0.812 | 68 | 1964 | <0.0001 | 0.858 |  |  |  |  |  |  |  |  |
|  | [1166-8979] | [7605-66213] |  |  | [5-330] | [397-8206] |  |  |  |  |  |  |  |  |  |  |
| **IL-10** | 5 | 23 | 0.0010 | 0.762 | 5 | 5 | 0.3093 | 0.557 |  |  |  |  |  |  |  |  |
|  | [5-15] | [8-55] |  |  | [5-5] | [5-5] |  |  |  |  |  |  |  |  |  |  |
| **IL-17A** | 5 | 5 | 0.0436 | 0.615 | 5 | 5 | >0.9999 | 0.511 |  |  |  |  |  |  |  |  |
|  | [5-5] | [5-5] |  |  | [5-5] | [5-5] |  |  |  |  |  |  |  |  |  |  |
| **IP-10** | 17505 | 63134 | 0.0003 | 0.788 | 1609 | 19224 | 0.0002 | 0.801 |  |  |  |  | 114 | 6227 | <0.0001 | 0.784 |
|  | [6466-21733] | [26551-100000] |  |  | [245-5383] | [2669-57398] |  |  |  |  |  |  | [10-679] | [338-34015] |  |  |
| **MIP-1α** | 47 | 773 | 0.0006 | 0.792 | 5 | 45 | <0.0001 | 0.868 |  |  |  |  |  |  |  |  |
|  | [22-241] | [84-6000] |  |  | [5-9] | [15-1556] |  |  |  |  |  |  |  |  |  |  |
| **sCD40L** | 5 | 30 | <0.0050 | 0.736 | 5 | 5 | 0.0769 | 0.639 |  |  |  |  |  |  |  |  |
|  | [5-12] | [5-59] |  |  | [5-6] | [5-21] |  |  |  |  |  |  |  |  |  |  |
| **TNF-α** | 31 | 276 | <0.0001 | 0.846 | 5 | 38 | <0.0001 | 0.868 | 10 | 38 | 0.0004 | 0.742 | 10 | 15 | 0.0018 | 0.703 |
|  | [23-51] | [107-893] |  |  | [5-5] | [7-115] |  |  | [10-16] | [10-62] |  |  | [10-12] | [10-36] |  |  |

Results of the measured concentrations are reported in pg/ml as medians and 25^th^ – 75^th^ percentiles. The degrees of significance of the differences between the concentrations measured in the two groups of subjects are reported as *p* values. The diagnostic ability of each cytokine was assessed by receiver operator characteristics (ROC) curve analysis and the areas under the curves are reported in the table. *Mann-Whitney U test
